# Investigations of immunogenic, allergenic and adjuvant properties of Cry1Ab protein after intragastric exposure in a food allergy model in mice

**DOI:** 10.1186/s12865-016-0148-x

**Published:** 2016-05-04

**Authors:** Monica Andreassen, Thomas Bøhn, Odd-Gunnar Wikmark, Johanna Bodin, Terje Traavik, Martinus Løvik, Unni Cecilie Nygaard

**Affiliations:** GenØk - Centre for biosafety, Tromsø, Norway; Norwegian Institute of Public Health, Oslo, Norway; UiT The Arctic University of Norway, Tromsø, Norway; North West University, Potchefstroom, South Africa; Norwegian University of Science and Technology, Trondheim, Norway; Present address: Department of Food, Water and Cosmetics, Norwegian Institute of Public Health, PO Box 4404, 0403 Oslo, Norway

**Keywords:** Food allergy, Anaphylaxis, Genetically modified maize, Cry1Ab, Mice

## Abstract

**Background:**

In genetically modified (GM) crops there is a risk that the inserted genes may introduce new allergens and/or adjuvants into the food and feed chain. The MON810 maize, expressing the insecticidal Cry1Ab toxin, is grown in many countries worldwide. In animal models, intranasal and intraperitoneal immunisations with the purified Cry1Ab proteins have induced immune responses, and feeding trials with Cry1Ab-containing feed have revealed some altered immune responses. Previous investigations have primarily measured antibody responses to the protein, while investigations of clinical food allergy symptoms, or allergy promotion (adjuvant effect) associated with the Cry1Ab protein are largely missing. We aimed to investigate immunogenic, allergenic and adjuvant properties of purified Cry1Ab toxin (trypCry1Ab, i.e., trypsin activated Cry1Ab) in a mouse model of food allergy.

**Method:**

Female C3H/HeJ mice were immunized by intragastric gavage of 10 μg purified, trypsin activated Cry1Ab toxin (trypCry1Ab) alone or together with the food allergen lupin. Cholera toxin was added as a positive control for adjuvant effect to break oral tolerance. Clinical symptoms (anaphylaxis) as well as humoral and cellular responses were assessed.

**Results:**

In contrast to results from previous airway investigations, we observed no indication of immunogenic properties of trypCry1Ab protein after repeated intragastric exposures to one dose, with or without CT as adjuvant. Moreover, the results indicated that trypCry1Ab given by the intragastric route was not able to promote allergic responses or anaphylactic reactions against the co-administered allergen lupin at the given dose.

**Conclusion:**

The study suggests no immunogenic, allergenic or adjuvant capacity of the given dose of trypCry1Ab protein after intragastric exposure of prime aged mice.

**Electronic supplementary material:**

The online version of this article (doi:10.1186/s12865-016-0148-x) contains supplementary material, which is available to authorized users.

## Background

The majority of food and feed products from genetically modified (GM) plants to date are claimed to be compositionally and nutritionally comparable to the parent crops (with the exception of the introduced traits) [1]. There is, however, a concern that the inserted genes may constitute a risk of introducing new allergens and/or adjuvants into the food and feed chain [2–5]. Food allergy is an important public health problem, with a prevalence of about 8 % in children and 3 % in adults [6]. In food allergic individuals, the normal development of oral tolerance has been disturbed, resulting in T helper type 2 (Th2) immune responses, IgE sensitization towards the allergen and a risk of immediate hypersensitivity reactions upon allergen re-exposure [7]. Adjuvants may contribute to this break of tolerance by a number of mechanisms such as increased cell surface expression of major histocompatibility complex (MHC) and co-stimulatory and/or adhesions molecules, and modulated release of cytokines [8, 9], although the mechanisms are not clarified in detail. The food allergic response involves several cell types and organ systems and can cause severe and sometimes fatal reactions. The primary treatment of food allergy is avoidance of the offending food items. Obviously, the introduction into the food chain of GM plants containing new food allergens or proteins with adjuvant potential is unwanted and an expressed concern [2].

A processed *cry1Ab-*gene from the soil bacterium *Bacillus thuringiensis* (Bt), coding for a bioactive Cry1Ab insecticidal toxin, has been inserted into the genome of the maize event MON810, in order to make these crops resistant to damage caused by lepidopterans. The Bt bacterium produces a Cry1Ab protoxin that will exert toxicity after activation by enzymatic cleavage in the gut of susceptible lepidopteran species [10]. In the MON810 plant, however, an already activated version of the toxin is expressed. The European Food Safety Authority (EFSA) has concluded that Cry1Ab-containing crops are regarded safe for human and animal consumption [8]. However, intranasal (i.n.) and intraperitoneal (i.p.) immunisations with the purified Cry1Ab proteins have been reported to elicit immune responses in mice [11] and we have previously demonstrated capacity of the trypsin activated Cry1Ab (trypCry1Ab) toxin to elicit specific immunoglobulin (Ig) E and IgG1 antibodies after i.n. exposure [12]. Furthermore, feeding trials have revealed inflammatory or immune responses related to the ingestion of Cry1Ab-containing feed in sensitised fish [13], weaning and old mice [14], rats [15] and pigs [16]. Previous assessments of Cry1Ab immune effects have mainly focused on its capacity to provoke cellular and/or humoral responses. The structurally similar Cry1Ac protein has demonstrated adjuvant capacity in several studies [17–19]. To our knowledge, only three studies have investigated allergic adjuvant effects of Cry1Ab, reporting no [20, 21] and possible [22] adjuvant capacity after exposure by airways installation, feeding and oral gavage, respectively. So far, no experimental studies have investigated whether Cry proteins have adjuvant properties in relation to clinical food allergy responses.

In the present work, we investigated whether repeated exposures to one high dose level of trypCry1Ab may promote allergic responses (i.e., act as adjuvant) in an anaphylactic food allergy model in mice. Based on the same immunization regime by intragastric (i.g.) exposure, we have also explored immunogenic and allergenic properties of trypCry1Ab by assessments of specific serum antibodies as well as intestinal gene expression.

## Results

### Assessment of adjuvant capacity of trypCry1Ab

#### Anaphylactic responses

**Fig. 1:**
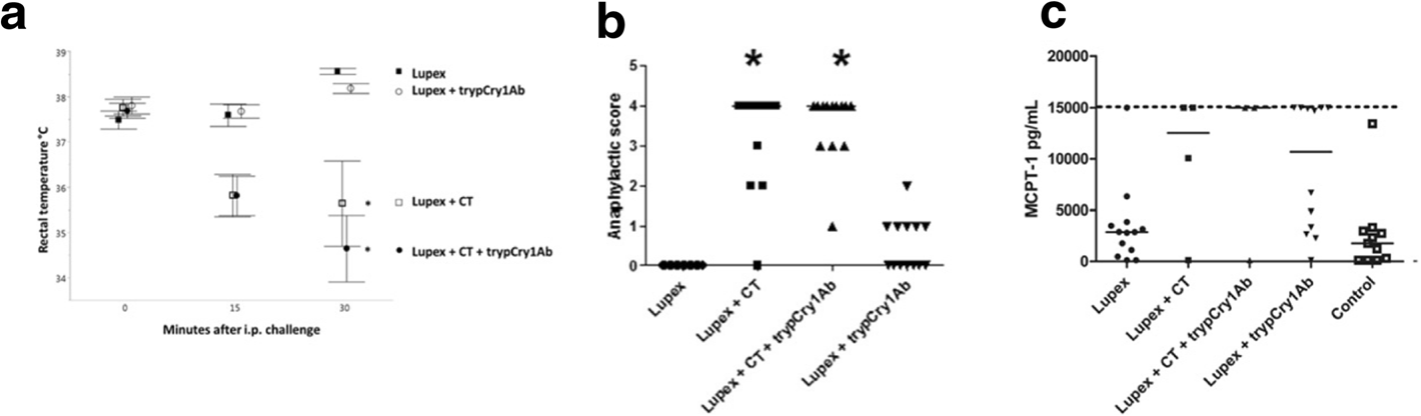
Anaphylactic responses in mice. Anaphylactic responses in mice after intragastric (i.g.) gavages with Lupex, Lupex + cholera toxin (CT), Lupex + CT + trypsinised Cry1Ab (trypCry1Ab), Lupex + trypCry1Ab, and HBSS (control) on days 0, 1, 2, 7, 21, and 28, followed by an intraperitoneal (i.p.) challenge with Lupex on day 35. Rectal temperature (**a**) was measured at 0, 15 and 30 min after challenge, and anaphylactic score (**b**) were determined continuously during those 30 min. Mouse mast cell protease-1 (MCPT-1) levels (**c**), a marker for intestinal anaphylaxis, was measured in serum collected after challenge (day 35). Temperatures are presented as group medians over time, while anaphylactic scores and serum MCPT-1 concentrations are presented for each individual animal (*dots*) and as the group median (*line*). *Asterisk* (*) denote groups that are significantly different (*p* < 0.05) from the Lupex group. The *dotted line* denotes the upper detection limit of the MCPT-1 assay

During the 30 min after i.p. challenge with allergen extract (Lupex) on day 35, the rectal temperature dropped significantly in mice i.g. immunised with Lupex + CT and Lupex + CT + trypCry1Ab compared to mice immunised with Lupex or Lupex + trypCry1Ab. There was no significant difference in rectal temperature between the Lupex + CT and Lupex + CT + trypCry1Ab immunised mice, or the Lupex and Lupex + trypCry1Ab immunised mice (Fig. 1a). Likewise, according to the anaphylactic score given during the 30 min observation period after the i.p. challenge with Lupex, the clinical response was significantly more pronounced in mice gavaged with Lupex + CT and Lupex + CT + trypCry1Ab, compared to mice gavaged with Lupex or Lupex + trypCry1Ab. The mice gavaged with Lupex + CT + trypCry1Ab did not display a significantly stronger anaphylactic response than mice gavaged with Lupex + CT, nor did the Lupex + trypCry1Ab versus Lupex exposed mice (Fig. 1b). Since animals experiencing strong anaphylactic shock (in particular Lupex + CT and Lupex + CT + trypCry1Ab) gave limited volume of blood at the terminal bleed, the number of blood samples per groups available for measurements of the anaphylaxis marker MCPT-1 were low (Fig. 1c). The few serum samples in these groups, however, all had high concentrations of MCPT-1, but this increase did not reach statistical significance relative to the two negative control groups due to the low sample numbers. Although not statistically significant for any of the endpoints, almost half of the animals in the Lupex + trypCry1Ab group tended to have a weak anaphylactic score and high MCPT-1 concentrations.

#### Serum levels of total IgE and lupin-specific antibodies on day 34

**Fig. 2:**
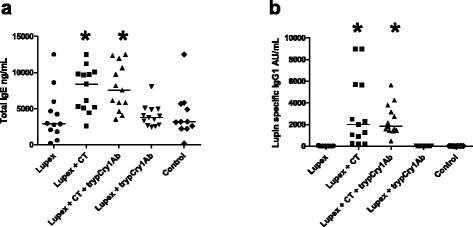
Serum levels of total IgE and lupin specific IgG1. Serum levels of total IgE (**a**) and lupin specific IgG1 (**b**) in mice after intragastric (i.g.) gavages with Lupex, Lupex + cholera toxin (CT), Lupex + CT + trypsinised Cry1Ab (trypCry1Ab), Lupex + trypCry1Ab, and HBSS (control) on day 0, 1, 2, 7, 21, and 28. Levels are determined in blood samples drawn at day 34. Results are presented for each individual animal (*dots*) and as the group median (*line*). *Asterisk* (*) denote groups that are significantly different (*p* < 0.05) from the Lupex group

The serum levels of both total IgE (Fig. 2a) and lupin-specific IgG1 (Fig. 2b) were significantly higher in mice gavaged with Lupex + CT and Lupex + CT + trypCry1Ab than in the control and Lupex groups, i.e., irrespective of the presence of trypCry1Ab. Neither did the antibody levels differ between groups receiving Lupex with or without trypCry1Ab.

#### Splenocyte secretion of cytokines

**Fig. 3:**
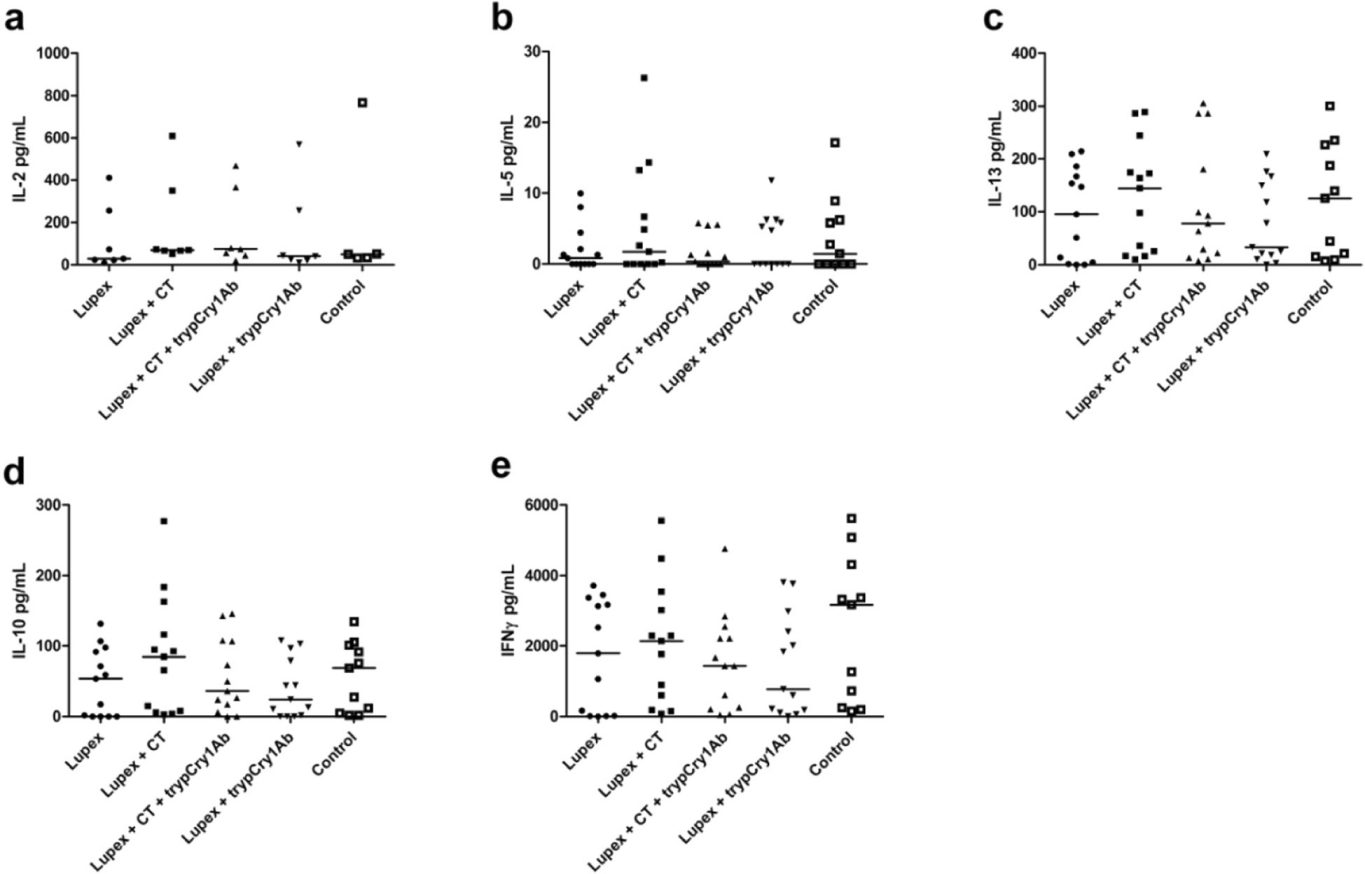
Cytokine levels in spleen cell supernates from Lupin exposed mice. Cytokine IL-2 (**a**), IL-5 (**b**), IL-13 (**c**), IL-10 (**d**) and IFNγ (**e**) levels in supernates of concanavalin A (ConA) stimulated spleen cells from mice intragastrically (i.g.) gavaged with Lupex, Lupex + cholera toxin (CT), Lupex + CT + trypsinised Cry1Ab (trypCry1Ab), Lupex + trypCry1Ab, and HBSS (control) on day 0, 1, 2, 7, 21, and 28, and intraperitoneal (i.p.) challenged with Lupex on day 35. Results are presented for each individual animal (*dots*) and as the group median (*line*)

After stimulation of cultured spleen cells with 17.2 μg/ml Lupex for five days, the supernates contained low levels or levels below the detection limit of the cytokines IL-2, -5, -13, -10 and IFNγ (data not shown). Stimulation with 5 μg/ml ConA for 72 h induced secretion of IL-2, -5, -13, -10 and IFNγ cytokines, however, the levels were not significantly different between the treatment groups (Fig. 3).

### Assessment of immunogenicity of trypCry1Ab

#### Serum levels of total IgE and Cry1Ab-specific antibodies

**Fig. 4:**
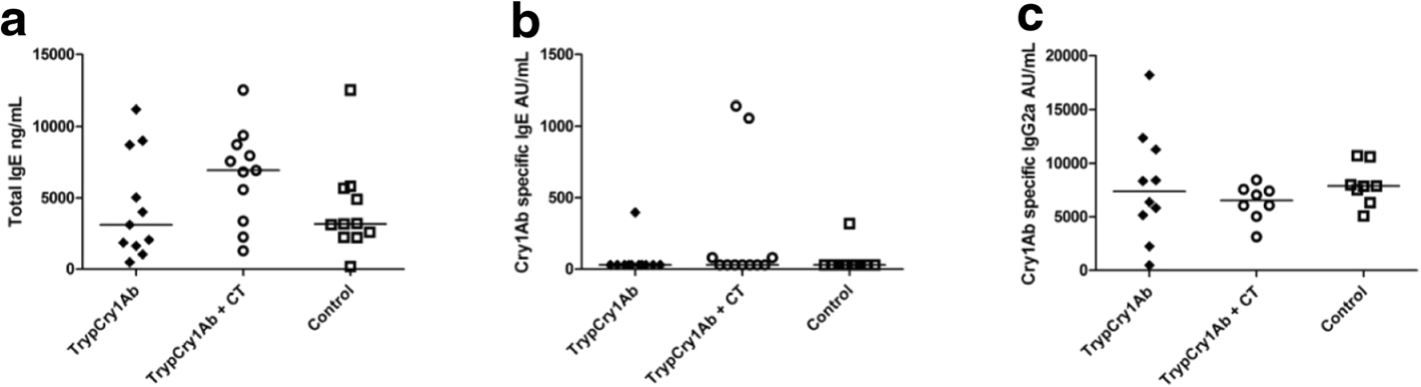
Serum levels of total IgE and Cry1Ab-specific IgE and IgG2a. Serum levels on day 34 of total IgE (**a**), Cry1Ab-specific IgE (**b**) and Cry1Ab-specific IgG2a (**c**) in mice receiving intragastric (i.g.) gavage with trypsinised Cry1Ab (trypCry1Ab), trypCry1Ab + cholera toxin (CT) and HBSS (control) on days 0, 1, 2, 7, 21, and 28. Results are presented for each individual animal (*dots*) and as the group median (*line*)

Serum levels of total IgE, Cry1Ab-specific IgE and IgG2a did not significantly differ between the groups exposed to vehicle (control), trypCry1Ab or trypCry1Ab + CT (Fig. 4). Cry1Ab-specific IgG1for all individuals were below the assay limit of detection (data not shown).

#### Splenocyte secretion of cytokines

**Fig. 5:**
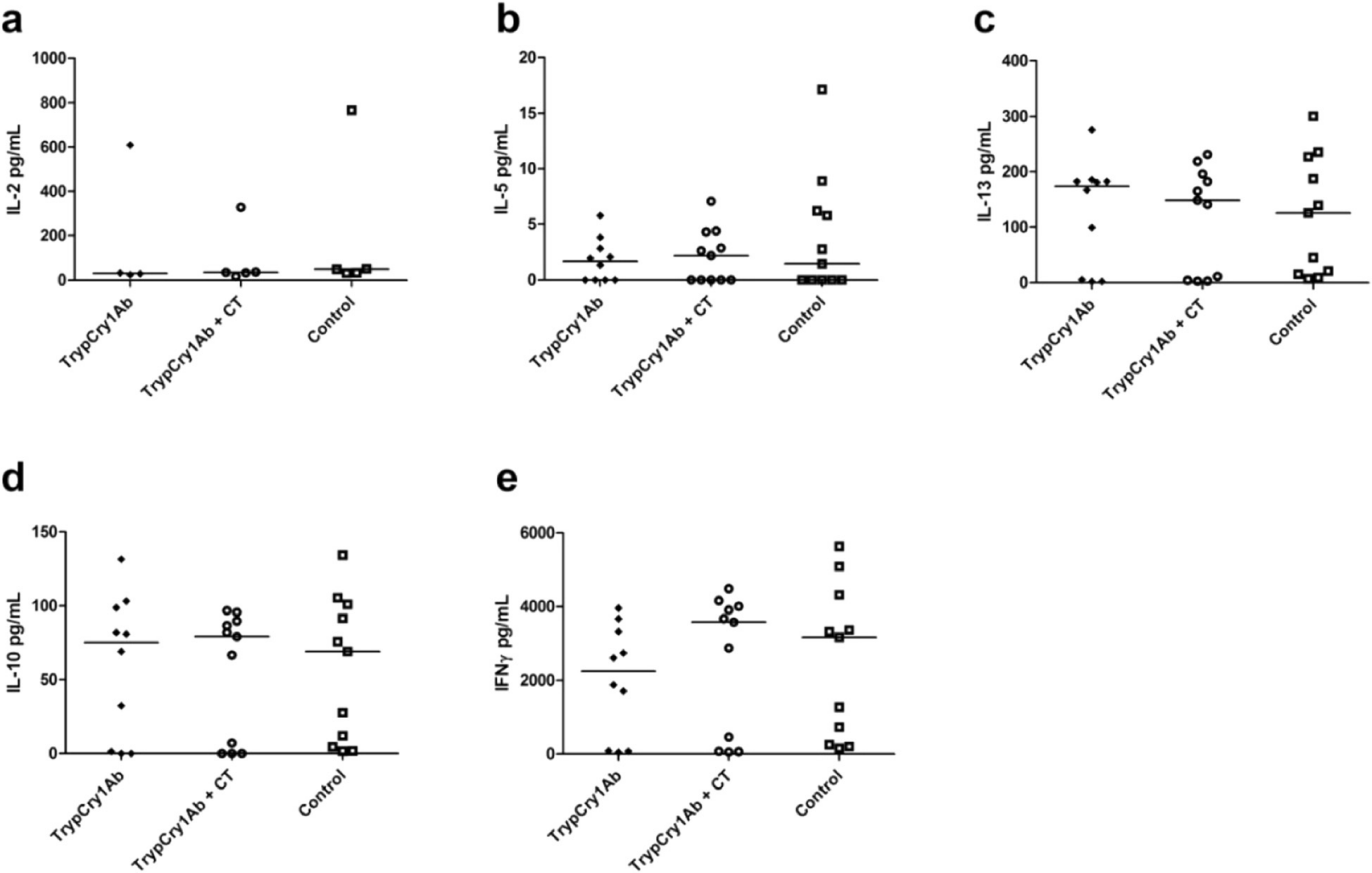
Cytokine levels in spleen cells supernates from Cry1Ab exposed mice. Cytokine IL-2 (**a**), IL-5 (**b**), IL-13 (**c**), IL-10 (**d**) and IFNγ (**e**) levels in supernates of concanavalin A (ConA) stimulated spleen cells from mice receiving intragastric (i.g.) gavage with trypsinised Cry1Ab (trypCry1Ab), trypCry1Ab + cholera toxin (CT) and HBSS (control) on days 0, 1, 2, 7, 21, and 28. Results are presented for each individual animal (*dots*) and as the group median (*line*)

Exposure to trypCry1Ab or trypCry1Ab + CT did not affect the IL-2, -5, -13, -10 and IFNγ cytokine levels secreted by ConA stimulated splenocytes compared to the negative control (Fig. 5). After splenocyte stimulation with 17.2 μg/ml Cry1Ab for five days, supernates contained low levels or levels below the detection limit of the cytokines (data not shown).

### Local effects: gene expression in the small intestine

**Fig. 6:**
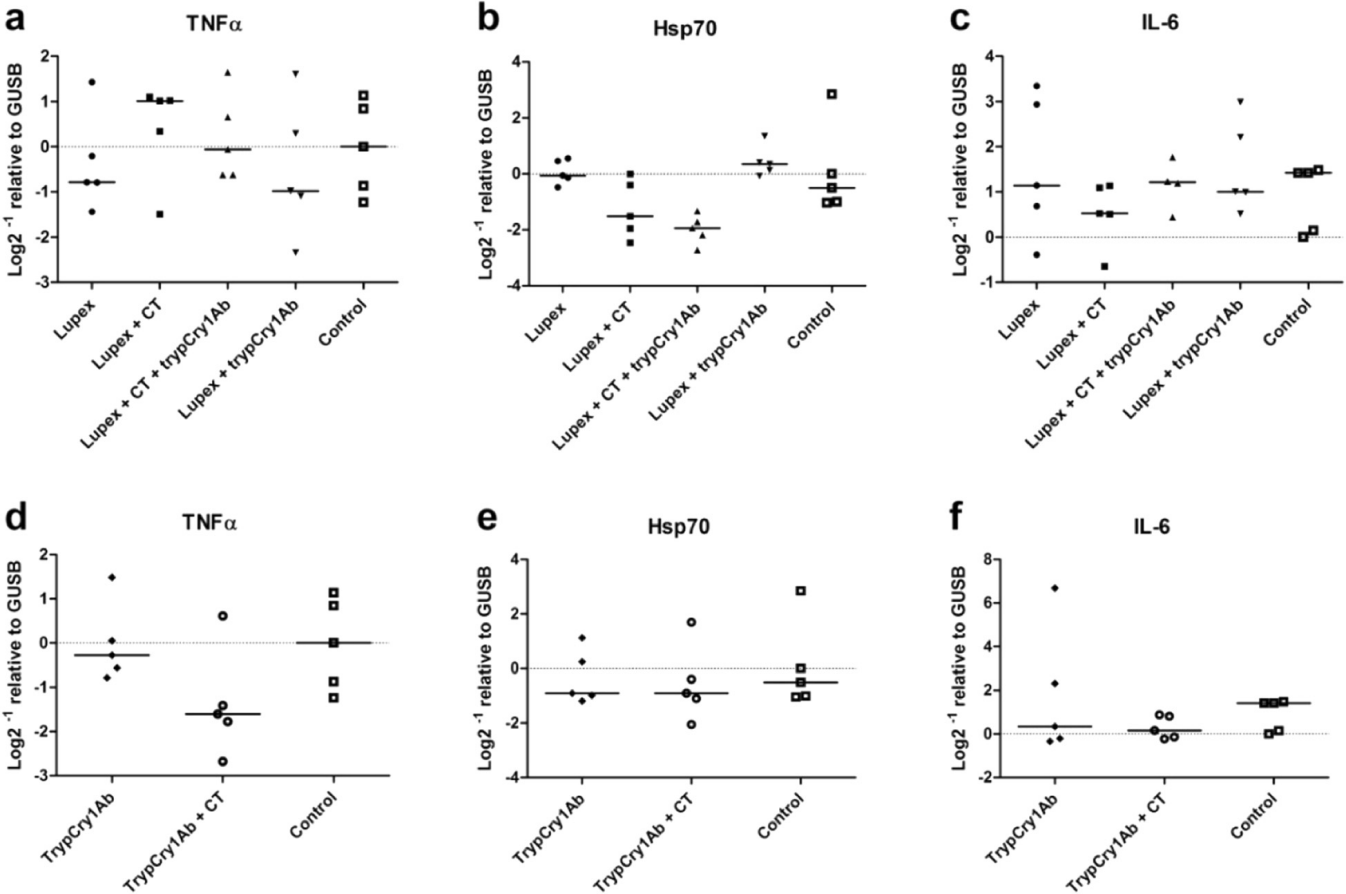
Gene expressions in the small intestine. Relative quantification of TNFα, HSP70 and IL-6 mRNA in the small intestine of mice receiving intragastric (i.g.) gavage with Lupex, Lupex + cholera toxin (CT), Lupex + CT + trypsinised Cry1Ab (trypCry1Ab), Lupex + trypCry1Ab, and HBSS (control) (**a**, **b**, **c**), and with trypCry1Ab, trypCry1Ab + CT and HBSS (control) (**d**, **e**, **f**). Results are presented for each individual animal (*dots*) and as the group median (*line*), and the dotted line denotes the 0-line i.e., no difference relative to the housekeeping gene GUSB

The expression of the genes HSP70, MCPT-1, IL-6, IL-9 and TNFα in tissue from the small intestines was determined at termination. Neither between the groups in the adjuvance set-up (Table 1, Fig. 6a, b, c) nor in the immunogenicity set-up (Fig. 6d, e, f) did expression of TNFα, HSP70 and IL-6 relative to GUSB (housekeeping gene) differ between the treatment groups. The expression of the genes MCPT-1 and IL-9 relative to GUSB were below the limit of detection (data not shown).

**Table 1:** Exposure schemes

Effect	Group	Test solutions i.g. Days 0, 1, 2, 7, 21, and 28	Challenge i.p. Day 35	Number of mice
Adjuvanticity	A	Lupex	Lupex	13
	B	Lupex + CT	Lupex	13
	C	Lupex + CT + trypCry1Ab	Lupex	13
	D	Lupex + trypCry1Ab	Lupex	13
Vehicle control	E	HBSS	-	11
Immunogenicity	F	TrypCry1Ab	-	11
	G	TrypCry1Ab + CT	-	11

*i.g.* intragastric, *i.p* intraperitoneal, *Lupex* lupin extract, *CT* cholera toxin

## Discussion

We investigated the capacity of a 10 μg dose of trypCry1Ab protein given repeatedly by peroral gavage to act as an adjuvant and/or to induce immune responses, in a mouse model of food allergy. The mucosal adjuvant CT with or without trypCry1Ab was able to condition for allergic sensitisation and thus anaphylactic responses in allergen-challenged mice, while trypCry1Ab at the given dose did not affect any of the allergy or anaphylaxis markers included in the adjuvance experiment. Furthermore, there was no indication of an immunogenic potential for i.g. administered trypCry1Ab in the model and with the dose employed.

We did not observe any adjuvant capacity of the trypCry1Ab protein on the clinical anaphylactic endpoints (i.e., loss in temperature and clinical score) or on sensitisation (total IgE and lupin-specific IgG1), neither when administered with Lupex with or without the tolerance-breaking CT. Serum MCPT-1, a marker of anaphylaxis in food allergy mouse models [23, 24], was increased in half of the animals exposed to Lupex + trypCry1Ab compared to the animals exposed to Lupex or vehicle (control). The elevated MCPT-1 in these few animals was, however, not accompanied with any changes in temperature loss, antibody or cytokine responses after challenge or IgE sensitisation, thus did not seem to be associated with an allergic or anaphylactic reaction. Further, no increase in MCPT-1 was observed in the groups exposed to trypCry1Ab and trypCry1Ab + CT (data not shown), indicating that trypCry1Ab itself was not eliciting a MCPT-1 response. Unfortunately, expression of the MCPT-1 gene was not detectable in the intestinal tissue, and cannot contribute to confirm or reject the observation in serum. Thus, it seems reasonable not to regard the increase in serum MCPT-1 in a few animals as an adverse outcome, although the possibility that trypCry1Ab with Lupex elicited local effects in the gastrointestinal tract cannot be excluded, also in light of previous reports [13]. We therefore conclude that the single dose of trypCry1Ab did not act as an adjuvant in the present experiments.

We have recently demonstrated that airway exposure to different versions of the trypCry1Ab protein did not promote allergic responses comparable to those observed with the inclusion of an adjuvant (CT) in mice sensitised with the allergen ovalbumin (OVA) [25]. Furthermore, a recent study by Reiner et al. [20] investigated the initiation and severity of allergic asthma in mice fed a MON810 containing diet, and they were not able to detect any differences in eosinophilic airway and lung inflammation, mucus hypersecretion or OVA-specific antibody production, compared to mice given a non-GM diet. Taken together with the previous study, our present data from a clinical food allergy model further support the notion that trypCry1Ab do not promote development of immune responses towards another allergen, i.e., exerts allergic adjuvant effect. These findings are also partly in agreement with a previous investigation of the adjuvant capacity of trypCry1Ab in a model studying lung effects in orally sensitized animals [22]. As in the present study, the study showed no detectable effects of Cry1Ab on sensitisation parameters after intragastric exposure. However, while we did not see any effect after i.p. allergen challenge, Guimaraes et al. reported that oral Cry1Ab enhanced local effects in the lungs after airway peanut challenge. Observed responses after airway allergen challenge were early production of leukotrienes in bronchoalveolar lavage fluid (BALF), and late Th2- and Th17-cytokine production and eosinophil/neutrophil influx. The methodological differences in doses, route of challenge and target organ (lungs versus intestines) do not allow for a direct comparison of results on local effects.

In the present study, the trypCry1Ab protein showed no indications of an immunogenic potential, even when administered together with a potent adjuvant, as the levels of Cry1Ab-specific antibody levels in serum were low (IgE and IgG2a) or below the limit of detection (IgG1) in mice repeatedly gavaged with a single dose of trypCry1Ab. In contrast, we previously demonstrated strong Cry1Ab-specific IgG1 and IgE responses following the i.n. route of exposure to a lower dose of trypCry1Ab [12]. Others have also detected the immunogenic potential of Cry1Ab proteins. For example, i.n. and i.p. immunisation with the trypCry1Ab protein induced production of specific antibody responses in mice in a study by Guerrero et al. [11]. Also, in rats fed transgenic Bt rice or feed spiked with the trypCry1Ab a significant elevation of specific antibodies was detected [26]. Notably, in that study, specific antibody responses were induced in all rats, including the control fed rats, suggesting that the airborne particles or dust from the Cry1Ab feed and/or the Cry1Ab spiked feed induced immune responses after inhalation. Hence, the Kroghsbo study cannot conclude on an immunogenic potential after intragastric exposure to Cry1Ab. Adel-Patient et al. [27] demonstrated that the i.p. route gave a mixed Th1/Th2 antibody response towards the purified Cry1Ab protoxin, while in contrast, i.g. administration of purified Cry1Ab protoxin or MON810 protein extract did not have any impact on the humoral immune response in mice. Thus, although previous investigations suggest that the Cry1Ab protein may be immunogenic after airway exposures, the present study support the previous observations that Cry1Ab proteins do not exert these properties after i.g. exposures. Because proteins that are able to escape the prototypical degradation in the digestive tract are more likely to reach the intestinal mucosa and to sensitise the mucosal immune system [7], stability to digestion is considered to be one of several tools to assess the allergenic potential of a protein [28]. Guimaraes et al. [29] used simulated gastric fluid (SGF) at different pH values and demonstrated that while Cry1Ab were highly degraded at pH 1.2, it contained its immunoreactivity at pH 2, suggesting that the specific gastric conditions may influence the outcome of allergenicity assessments. Nevertheless, degradation in the intestinal tract may explain why immunogenicity seldom is reported after feeding or i.g. studies, while other administration routes not involving similarly efficient degradation cause cellular and humoral responses.

Induction of allergic immune responses following mucosal immunisation is usually dependent on the co-administration of appropriate adjuvants to achieve the allergy-associated Th2 response [30]. In our food allergy model this is demonstrated by the necessity for the allergen to be co-administered with CT to disrupt the oral tolerance and thus enhance antibody responses (Fig. 2) and the anaphylactic reactions (Fig. 1) against the Lupex allergen. For investigations of adjuvant effects, we chose two approaches. Firstly, to test whether the trypCry1Ab protein could act as an adjuvant with similar potency as CT, CT was replaced with the trypCry1Ab protein. Secondly, to test whether trypCry1Ab could act as a further adjuvant in susceptible individuals where the oral tolerance already is broken, we also exposed animals to Lupex + CT + trypCry1Ab. The latter would allow detection of adjuvants in situations where other circumstances (i.e., exposure to other immunotoxic components like particles [31, 32], gastrointestinal infections or genetic susceptibilities are also in favor of allergy induction, as for instance reported by Bol-Shoenmakers et al. [33]. Since this latter approach would also allow detection of weaker adjuvants, the negative results strengthen the conclusion that the given dose of trypCry1Ab does not present adjuvant activity after oral exposure.

The use of the mucosal adjuvant CT in assessment of allergenicity of novel proteins, however, has been debated. Atkinson and Miller [34] suggest that the use of CT as adjuvant may compromise the discrimination between proteins with respect to allergenic potential. We included the mucosal adjuvant CT together with the trypCry1Ab protein (Figs. 4, 5 and 6) to disrupt the oral tolerance, which as described above may happen in real life. In line with the criteria suggested by Prescott and Hogan [4] for the assessment of GM foods we also included an experimental group without the CT. Overall, neither of the experimental groups exposed to trypCry1Ab differed from the respective control group, strengthening our conclusion of no immunogenic or adjuvant properties of the trypCry1Ab proteins at the given dose after i.g. exposure.

In the food allergy models by Vinje et al. [23] and Li et al. [35], IgE antibodies against lupin and peanut allergens, respectively, were detected in the serum of female C3H/HeJ mice, and splenocytes demonstrated a mixed Th1/Th2 cytokine release and proliferative responses after ex vivo stimulation with allergens in the form of extracts or purified proteins. In addition, serum MCPT-1 together with high anaphylactic scores indicated the usefulness as a mouse model for clinical food allergy. In agreement, the positive control in the present study, Lupex + CT, also showed increased antibody, MCPT-1 and anaphylactic responses. On the other hand, the cultured spleen cells stimulated with the antigens (lupin or trypCry1Ab) secreted low or undetectable levels of the cytokines IL-2, IL-5, IL-13, IL-10 and IFNγ. In the cell cultures, the antigens were added at much lower dose than in the original protocol by Vinje et al. [23] (17,2 versus 50 μg) due to practical limitations in the solubility of trypCry1Ab proteins, which probably explain why cytokine release were not elevated even in the positive control (Lupex + CT). Spleen cells stimulated with ConA (5 μg/ml), as an “unspecific” T cell stimulating mitogen probably stimulating a much higher proportion of the T cells, secreted IL-2, IL-5, IL-13, IL-10 and IFNγ cytokines, however these levels were not different between any of the treatments. A trend, however not significant, was detected in the positive control (Lupex + CT) and it may be speculated that the time interval from the latest exposure on day 28 until the harvest and stimulation of splenocytes on day 35 did not allow for a robust secretion of cytokines.

To further look for local effects of Cry1Ab in the intestinal mucosa we assessed the expression of genes suggested to be involved in the immune response in the intestinal tissue, as indicated in a study by Gu et al. [13]. The expression levels were quantified by normalisation against GUSB, identified as one of the most stable reference genes in the mouse small intestine, also compared to the commonly used reference gene glyceraldehyde 3-phosphate dehydrogenase (GAPDH) proving to be unstable [36]. The present data could not confirm the alterations in gene expression reported in Gu and coworkers’ study, where feeding of MON810 maize seemed to potentiate oxidative cellular stress in the intestine of sensitised fish, as indicated by increases in superoxide dismutase and HSP70 mRNA expression. We cannot exclude that the different species, exposure regime, timing (in our study it was three days since the last Cry1Ab exposure), or methodological considerations contributed to the observed lack of local gene expression changes in our mouse model.

The present study does not indicate adverse health effects of trypCry1Ab in terms of immunogenic, allergenic or adjuvant properties, however, there are several experimental limitations that should be considered. The Cry1Ab protein is expressed at low levels in plant material (6.92 and 612.51 ng/mg Cry1Ab in pollen and leaves, respectively, reported in Andreassen et al. [21]), and methods for isolation/extraction of plant Cry1Ab protein in sufficient amounts for this study was not available. Therefore, a recombinant version was produced in the *E. coli* bacteria and subsequently trypsin digested to “mimic” the activated form expressed in the plants. This introduces, however, a restriction to our conclusions, since trypCry1Ab toxin may have a different immunomodulation capacity than the toxin expressed in plant material due to subtle structural differences. Moreover, oral gavage was chosen as a model for oral exposure via food, and gives more accurate dose administrations than exposure via food. However, the daily bolus administration differs from the real-life situations where the proteins are consumed in whole foods. Furthermore, our results from a repeated oral gavage of one dose level of trypCry1Ab cannot directly be used to generalise for other doses (or exposure frequencies). Quantitative maize kernel Cry1Ab-expression data [37] indicates that the applied dose of 10 μg per mouse per gavage equals the amount of Cry1Ab expressed in 40 g of maize kernels, which represent a relatively extreme intake for these mice that typically consume 4–5 g feed per day. This high dose was chosen for a proof-of-principle approach, and since there were no effects observed at this dose level, no further experiments with lower doses were performed. As always, caution should be taken to directly transfer results from mice models to humans. Future research concerning Cry1Ab hazard characterisation, however, would benefit from use of the plant version of the Cry1Ab, several doses and/or realistic exposure regimes. Epidemiological investigations of GMO-safety in general, and immune system effects in particular, are sparse, and systematic studies are warranted.

## Conclusions

A single test model should not alone be used to prove or disprove the potential of GM proteins to induce adverse immune responses. However, taken together, the present study supports previous findings suggesting no detectable immunogenic, allergenic or adjuvant capacity of the trypCry1Ab protein after i.g. exposure, within the limitations of the model, the doses and the protein contexts applied.

## Methods

### Animals

Female C3H/HeJ mice (Jackson Laboratories, USA), 5 weeks old at arrival, were randomly allocated to groups (*n* = 11–13) and housed on Nestpack bedding (Datesand Ltd, Manchester, UK), 3–5 mice in each cage. The Harlan Teklad 2018 rodent diet and tap water were given *ad libitum*. The mice were exposed to a 12/12 h light/dark cycle, room temperature (RT) of 21 +/− 2 °C and 55 +/− 10 % humidity. The experiment was performed in conformity with the laws and regulations for live animals in Norway, and approved by the Norwegian Animal Research Authority under the Ministry of Agriculture (FOTS ID 4534).

### TrypCry1Ab protein

TrypCry1Ab toxin was purchased from Case Western Reserve University, (Ohio, USA, Dr. Marianne Pusztai-Carey). The origin of the *cry1Ab* gene inserted in *Escherichia coli* (*E. coli*) was the *Bt kurstaki HD-1* strain. The inclusion bodies were solubilised at pH 10.5 in the presence of a reducing agent and the precipitated protoxins were digested by commercial bovine trypsin and subsequently purified by ion exchange high-performance liquid chromatography (HPLC). The relevant fractions were analysed by gel filtration, HPLC and sodium dodecyl sulphate polyacrylamide gel electrophoresis (SDS-PAGE), desalted and lyophilised (M. Carey, personal communication). The protein preparations were dissolved in a sterile physiological buffer (Hanks Balanced Salt Solution; HBSS) to a concentration of 172 μg/ml.

### Lupin extract and cholera toxin (CT)

Protein extract from *Lupinus angustifolius* (Lupex) was produced and provided by The National Veterinary Institute of Norway. The extract preparation procedure is described in Vinje et al. [23], and the protein extract had a concentration of 72.4 mg/ml. Cholera toxin (CT) from *Vibrio cholerae,* azide free, 1 mg/ml solution, was purchased from Quadratech Diagnostics Ltd (Surrey, UK).

### Preparation of test solutions

The test solutions were prepared in HBSS and given to the mice in volumes of 250 μl, resulting in the following doses per animal per gavage: A; 5.7 mg Lupex, B; 5.7 mg Lupex + 10 μg CT, C; 5.7 mg Lupex + 10 μg CT + 10 μg trypCry1Ab, D; 5.7 mg Lupex + 10 μg trypCry1Ab, E; 10 μg trypCry1Ab, F; 10 μg trypCry1Ab + 10 μg CT. The control solution G consisted solely of 250 μl HBSS. A high dose of 10 μg trypCry1Ab was selected due to the proof-of-principle character of the study and in order to be able to compare a potential adjuvant effect with the effect induced by 10 μg of the positive control, CT. Restrictions in gavage volume (max 250 ml) and the trypCry1Ab solubility prevented us from giving a higher dose per animal per exposure.

### Experimental design

The mice were exposed by i.g. gavage of 250 μL test solutions according to Table 1, on days 0, 1, 2, 7, 21, and 28. A 100 μL blood sample was collected from *vena saphena lateralis* from each animal on day 0 and 34. On day 35, to examine the potential adjuvant effect of trypCry1Ab, a challenge of Lupex was given i.p. to all mice in the experimental groups A - D according to Table 1, and the anaphylactic responses were assessed during 30 min following the i.p. challenge (described below). All mice were anaesthetised with 3.5 % Isofluran gas (Isoba vet; Intervet/Schering-Plough Animal Health, Lysaker, Norway) administered in surgical O_2_ in an inhalation chamber before exsanguination by heart puncture, and blood, spleen and 5 cm of the small intestine (distal ileum) were harvested.

### Assessment of clinical anaphylactic reactions

On day 35, clinical anaphylactic reactions were assessed continuously in 30 min directly after an i.p. challenge with 250 μl of HBSS containing 5 mg of the allergen. The score system described by Li et al. [35] was employed: 0, no symptoms; 1, scratching and rubbing around the nose and head; 2, puffiness around the eyes and mouth, diarrhoea, pili erecti, inactivity or decreased activity with an increased respiratory rate; 3, wheezing, laboured respiration, cyanosis around the mouth and tail; 4, no activity after prodding or tremor or convulsion; and 5, death. Rectal temperature was measured after 0, 15 and 30 min with a BAT-12 microprobe thermometer (probe RET-3) purchased from Physitemp Instruments Inc (Clifton, USA). The mice were exsanguinated by terminal heart bleed immediately after the 30 min observation period, or when reaching a score of 4 or above.

### Measurement of serum mouse mast cell protease-1 (MCPT-1)

As a marker of anaphylaxis, serum levels of mouse mast cell protease-1 (MCPT-1) were analysed in terminal blood samples collected after allergen challenge, with an enzyme-linked immunosorbent assay (ELISA) kit (Moredun Scientific Ltd., Scotland, UK) in accordance with the manufacturer’s instructions.

### Detection of lupin-specific antibodies

The detection of lupin-specific IgG1 antibodies was performed according to Vinje et al. [23]. In brief; microtiter plates with 96 wells were coated with lupin extract (5 μg/ml), incubated for 1 h at room temperature (RT) and then overnight at 4 °C. After washing (Tris/Tween), blocking (Tris/Tween with 1 % bovine serum albumin) for 1 h, and subsequent washing, plates were incubated for 2 h at RT with lupin-specific IgG1 standard serum in fourfold dilutions for standard curve generation, negative control serum and the sera collected at day 34 (diluted 1:100 in BSA/Tris/Tween). After washing, peroxidase-labelled rat anti-mouse IgG1 antibody was added to each well. Following 2 h incubation at RT, and washing, plates were incubated for 15 min with peroxidase substrate. Plates were read at 405 nm in a BioTek Elx808 Absorbance Microplate reader using Gen5™ Microplate Data Collection & Analysis Software (BioTek® Instruments, Inc., Winooski, Vermont, USA).

### Detection of Cry1Ab-specific antibodies

*In-house* ELISA protocols were established to detect specific anti-Cry1Ab IgG1, IgG2a and IgE antibodies in the mouse sera as previously described in Andreassen et al. In brief, for the IgG1 assay, 96-well microtiter plates were coated with 2 ng/μL purified trypCry1Ab protein per well and incubated for 1 h at RT and then overnight at 4 °C. Plates were washed (Tris/Tween) and incubated with blocking solution (5 % skimmed milk powder in phosphate buffered saline (PBS)) for 1 h at RT. After subsequent washing, diluted sera (1:50) from terminal blood samples were added and plates were incubated for 1 h at RT and again overnight at 4 °C. The plates were washed, 200 ng biotinylated rat anti-mouse IgG1 was added per well and plates were incubated for 1 h at RT. After subsequent washing, poly-horseradish peroxidase (HRP)-streptavidin diluted 1:40000 was added and incubated for 1 h at RT. The plates were washed and color development was obtained by adding stabilised chromogen 3,3′, 5,5;-tetramethylbenzidine (TBM). After incubation in darkness for maximum 15 min the reaction was stopped with 2 N H_2_SO_4_ solution.

For detection of Cry1Ab-specific IgE, microtiter plates with 96 wells were coated with 200 ng rat anti mouse IgE antibodies per well, incubated for 1 h at RT and then overnight at 4 °C. Subsequently, the plates were washed with Tris/Tween and blocked with 5 % skimmed milk in PBS for 1 h at RT. After subsequent washing, diluted sera (1:10) from blood sampled at day 34 were added and plates were incubated for 1 h at RT and then again overnight at 4 °C. The plates were washed, 300 ng trypCry1Ab was added to each well and incubated for 1 h at RT. After subsequent washing, 300 ng biotinylated rabbit anti mouse Cry1Ab antibody was added per well as detection antibody and plates were incubated for 1 h at RT. Plates were washed and detection was performed with poly-HRP-streptavidin and stabilised chromogen TBM as described above. The reaction was stopped with 2 N H_2_SO_4_ solution after incubation in the dark for a maximum of 15 min.

For detection of Cry1Ab-specific IgG2a, microtiter plates with 96 wells were coated with 200 ng biotinylated rat anti mouse IgG2a antibodies per well, and the protocol for Cry1Ab-specific IgE detection was subsequently followed as described above. To accelerate the reactivity of each step, all incubations were performed at 37 °C for the detection of specific anti-Cry1Ab IgG2a.

Standard curves were made from duplicates of diluted serum pools from mice (repeatedly i.p.) immunised with trypCry1Ab and Al(OH)_3_, and included for all antibody assays on each plate. As the amount of specific IgG1, IgE and IgG2a in the standards is unknown, the levels are presented as arbitrary units (AU) per ml serum. Absorbance was measured at 450 nm on a BioTek Elx808 Absorbance Microplate reader with the Gen5™ Microplate Data Collection & Analysis Software (BioTek® Instruments, Inc., Winooski, Vermont, USA).

### Total IgE

Serum levels of total IgE were analysed in blood samples from day 34, using a Mouse IgE ready-set-go ELISA-kit (Affymetrix eBioscience, Vienna, Austria) in accordance to the manufacturer’s instructions.

### Splenocyte preparation and cytokine measurement

The excised spleens were crushed through a 70 μm cell strainer (BD labware, New Jersey, USA) to obtain single cell suspensions as described previously [38]. Cell numbers were determined with a Cell Coulter Z1 (Beckman Coulter Inc., Florida, USA). Cells were cultured in medium (RPMI 1640 with L-glutamine and 10 % foetal bovine serum and 1 % streptomycin/penicillin) in 96 well plates at a concentration of 2.7 × 10^6^ cells/ml per well at 37 °C in a humidified atmosphere with 5 % CO_2_ for three days with concanavalin A (ConA) (5 μg/ml), and for five days with or without Lupex or trypCry1Ab (17.2 μg/mL). The total volume in each well was 200 μl. The amount of cytokine IL-2, IL-5, IL-13, IL-10, and interferon gamma (IFNγ) released into spleen cell supernates were determined by Cytometric Bead Array (CBA) flex set kit from BD Biosciences (San Diego, California, USA).

### Gene expression in the small intestine

A 5 cm segment of ileum were excised (7 to 12 cm from the cecum), flushed thorough with PBS, cut into smaller fractions and put in a cryogenic vial that was snap frozen in liquid nitrogen and kept at –80 °C for later preparation and quantitative polymerase chain reaction (PCR) analyses. Frozen tissue segments were dissected with a scalpel on dry ice. Tissue segments between 14.3 and 68.8 mg were homogenised in a tube containing hard tissue CK28 beads (Bertine technologies, France) and 250 μL lysis buffer with 1.75 μL β-mercaptoethanol (Absolutely RNA Miniprep Kit, Agilent Technologies La Jolla, California), in a Precellys 24 homogeniser (Bertine Technologies, France) for 2 × 46 s at RT. Total RNA was extracted using Absolutely RNA Miniprep Kit (Agilent Technologies La Jolla, California) according to the manufacturer’s instructions. To assess yield and quality, the total RNA extract was evaluated using a Nandodrop spectrophotometer (Thermo scientific Wilmington, Denver, USA) and subsequently the RNA Integrity Number (RIN) was determined using a 2100 Bioanalyzer (Agilent Technologies La Jolla, California). Samples with RIN number lower than 4.8 were considered to be of poor quality and were not included in the following steps. First-strand cDNA synthesis of 50 ng total RNA were performed using a master mix containing 1xfirst strand MM buffer, 225 ng oligo(dT), 45 ng random hexamer and AffinityScriptRT/RNase block enzyme mixture (1.5 μL/30 μL total volume) and distilled water to a total volume of 30 μL (Agilent Technologies La Jolla, California). The syntheses were performed with a Bio Rad S1000 Thermal Cycler (Bio Rad Laboratories California, USA). The syntheses condition was 25 °C for 5 min, 42 °C for 30 min, 95 °C for 5 min and 12 °C for infinite. Real-Time PCR was performed by adding 1.0 μL cDNA solution to a master mix containing 1xPrimeTime assay (containing a probe and two primers), 1xBrilliant III Ultra-Fast QPCR Master Mix, 0.45 ng Reference dye and water to a total volume of 20 μL (Agilent Technologies La Jolla, California). The real-time PCR was performed with an Applied Biosystems 7500 Fast Real-Time PCR Systems (Applied Biosystems life technology, Thermo Scientific Waltham, Massachusetts, USA). The amplification conditions were 95 °C for 3 min, followed by 40 cycles at 95 °C for 20 s and 60 °C for 30 s. In total there were seven PrimeTime assays, five target genes; heat shock protein (HSP) 70 (primer 1: 5’-GTAGTACACAGTGCCAAGACG-3’, primer 2: 5’-TTTATATCAGTGTTCCAGTAGCCT-3’), MCPT-1 (primer 1: 5’-ACTCAACACCACCAATAATCTCC-3’, primer 2: 5’-GGAACCAGGACAAGAACACA-3’), IL-6 (primer 1: 5’-CAAGTGCATCATCGTTGTTCA-3’, primer 2: 5’-GATACCACTCCCAACAGACC-3’), IL-9 (primer 1: 5’-GCAGCTGGTCACGTTGC-3’, primer 2: 5’-CTTGCCTCTGTTTTGCTCTTC -3’), and tumour necrosis factor (TNF)α (primer 1: 5’-TCTTTGAGATCCATGCCGTTG-3’, primer 2: 5’-AGACCCTCACACTCAGATCA-3’), and two reference genes; TATA box binding protein (Tbp) (primer 1: 5’-CTGAATAGGCTGTGGAGTAACTC-3’, primer 2: 5’-CTGAAGAAAGGGAGAATCATGGA-3’) and glucuronidase-beta (GUSB) (primer 1: 5’-GATGCGTCTTATACCAGTTCTCA-3’, primer 2: 5’-CAACGCCAAATATGATGCAGAC-3’). The threshold cycle number (C_t_) was determined with ABI 7500 software from Applied Biosystems life technology (Thermo Scientific Waltham, Massachusetts, USA). Gene expression levels were normalised by subtraction of the housekeeping gene GUSB C_t_ from target gene C_t_, and then against the calibrator (= ΔΔC_t_). To obtain positive values for upregulated genes and negative values for downregulated genes, all ΔΔC_t_ values were multiplied with −1.

### Statistical analyses

Statistical analyses were performed with Sigma Plot 12.3 and Minitab 16 Statistical software. All normally distributed data with equal variance were tested by one way ANOVA. If significant overall differences were found, pairs of treatments were then tested with a Tukey adjusted post hoc test. Data that could not be transformed to normal distribution and comparable variance by log10 transformation were tested with the non-parametric Kruskal-Wallis test, while the Mann–Whitney post hoc test was used to evaluate differences between pairs of treatments. Differences between treatments were considered significant when *p*-values were below 0.05.

### Ethics approval

The experiment was performed in conformity with the laws and regulations for live animals in Norway, and approved by the Norwegian Animal Research Authority under the Ministry of Agriculture (FOTS ID 4534).

### Availability of data and material

The datasets supporting the conclusions of this article are included within the article (and its Additional file 1 “FOTS 4534_all datasets”).

## Additional file

Additional file 1: The Excel datafile “FOTS 4534_all datasets” includes all datasets supporting the conclusions of this article: Clinical endpoints, serum MCPT-1, serum total IgE, serum lupin-specific IgG1, splenocyte IL-2, IL-5, IL-10, IL-13 and IFNγ, serum Cry1Ab-specific IgE, IgG1 and IgG2a, and gene expression of TNFα, Hsp70 and IL-6 in the small intestine. (XLSX 124 kb)

## Abbreviations

Bt: *Bacillus thuringiensis*
CT: cholera toxin
GM: genetically modified
i.g.: intragastric
Lupex: lupin extract
trypCry1Ab: trypsin activated Cry1Ab
